# Pro- and Antioxidant Effects of Vitamin C in Cancer in correspondence to Its Dietary and Pharmacological Concentrations

**DOI:** 10.1155/2019/7286737

**Published:** 2019-12-24

**Authors:** Elzbieta Pawlowska, Joanna Szczepanska, Janusz Blasiak

**Affiliations:** ^1^Department of Orthodontics, Medical University of Lodz, 92-216 Lodz, Poland; ^2^Department of Pediatric Dentistry, Medical University of Lodz, 92-216 Lodz, Poland; ^3^Department of Molecular Genetics, Faculty of Biology and Environmental Protection, University of Lodz, 90-236 Lodz, Poland

## Abstract

Vitamin C is an antioxidant that may scavenge reactive oxygen species preventing DNA damage and other effects important in cancer transformation. Dietary vitamin C from natural sources is taken with other compounds affecting its bioavailability and biological effects. High pharmacological doses of vitamin C may induce prooxidant effects, detrimental for cancer cells. An oxidized form of vitamin C, dehydroascorbate, is transported through glucose transporters, and cancer cells switch from oxidative phosphorylation to glycolysis in energy production so an excess of vitamin C may limit glucose transport and ATP production resulting in energetic crisis and cell death. Vitamin C may change the metabolomic and epigenetic profiles of cancer cells, and activation of ten-eleven translocation (TET) proteins and downregulation of pluripotency factors by the vitamin may eradicate cancer stem cells. Metastasis, the main reason of cancer-related deaths, requires breakage of anatomical barriers containing collagen, whose synthesis is promoted by vitamin C. Vitamin C induces degradation of hypoxia-inducible factor, HIF-1, essential for the survival of tumor cells in hypoxic conditions. Dietary vitamin C may stimulate the immune system through activation of NK and T cells and monocytes. Pharmacological doses of vitamin C may inhibit cancer transformation in several pathways, but further studies are needed to address both mechanistic and clinical aspects of this effect.

## 1. Introduction

Vitamin C (ascorbic acid, ascorbate) is an essential micronutrient that must be delivered either with the diet or as a supplement as humans lost the ability to synthesize it due to mutations in the gene encoding a terminal enzyme in the vitamin C biosynthetic pathway [1]. Vitamin C plays a role in many processes as a cofactor for enzymes involved in processes and effects important for cancer transformation: antioxidant defense, transcription, and epigenetic regulation of gene expression (see [2] for review). Vitamin C is also reported to exert beneficial effects in the immune system and inflammation, which is crucial in fighting precancerous and cancer cells by the host (reviewed in [3]). Anticancer potential of vitamin C is suggested by the results of many other laboratory studies on experimental animals and cell cultures (reviewed in [4]).

**Figure 1:**
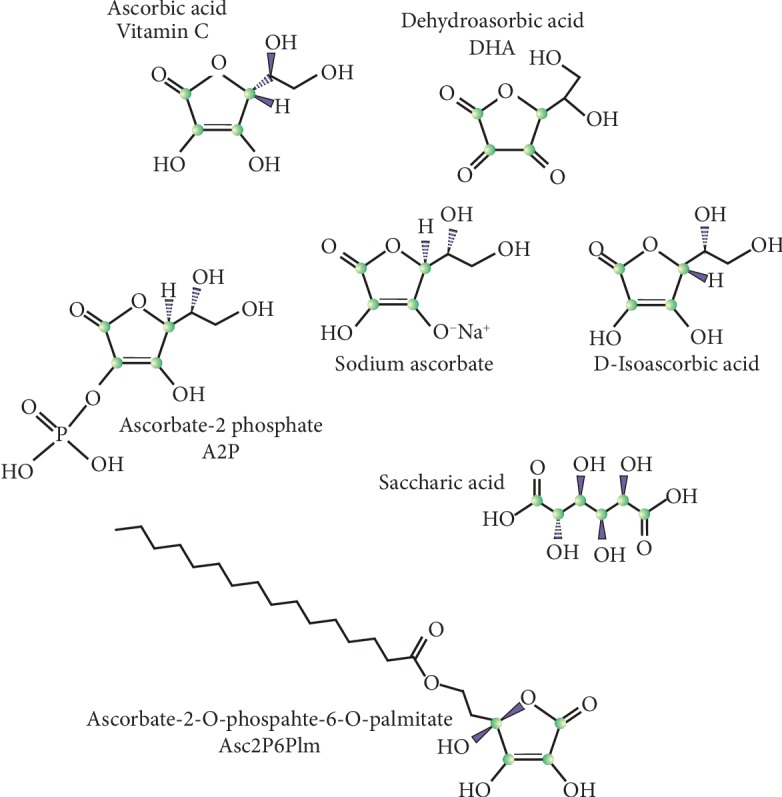
Molecular structure of vitamin C and its derivatives displaying anticancer properties that are discussed in this work.

Not only vitamin C but also its derivatives, including compounds with increased lipophilicity and resistance to oxidation, are used in anticancer studies (Figure 1).

Recently, van Gorkom et al. presented a systematic review on therapeutic application of vitamin C in cancer patients [5]. These authors reviewed 19 papers on the clinical use of vitamin C in patients with various malignancies in different settings, but they did not draw a single, definite conclusion on the efficacy of vitamin C in cancer therapy. This important work pointed out the low quality of many studies performed so far and their multiple weak points. Therefore, at present, the question on the clinically relevant positive effects of vitamin C in cancer is still open, but due to the importance of this problem and relative safety of the vitamin use, studies addressing it should be continued. Research on anticancer effects of vitamin C in randomized clinical trials should be preceded by a careful setting of clinical design and choice of endpoints to be evaluated.

Anticancer properties of vitamin C have been reviewed in several recent papers (e.g., [4, 6–10]). These studies suggest several potential targets of anticancer action of vitamin C—some of them will be described and referenced in the next sections. Five main vulnerabilities that can be targeted by vitamin C are redox imbalance, epigenetic reprogramming, oxygen sensing regulation, host immunity, and collagen synthesis in regard to metastasis. This manuscript focuses on molecular aspects of anticancer action of vitamin C, updates some information contained in those reviews, and distinguishes between dietary and pharmacological vitamin C in preventive and therapeutic interventions. A short note of natural versus synthetic vitamin C is made.

## 2. Bioavailability: Natural versus Synthetic Ascorbic Acid

The bioavailability of vitamin C ingested into the body is its proportion, which reaches systemic circulation and thus becomes available for physiological metabolic processes. When dietary vitamin C is ingested, a fraction of it is absorbed by the intestines. Vitamin C is actively transported within the body by two sodium-dependent transporters—SVCT1 and SVCT2—that display different tissue specificity and kinetics of uptake [11]. An oxidized form of vitamin C, dehydroascorbate (DHA), can be taken up by glucose transporters GLUT1-3 and GLUT8 [12–14].

**Figure 2:**
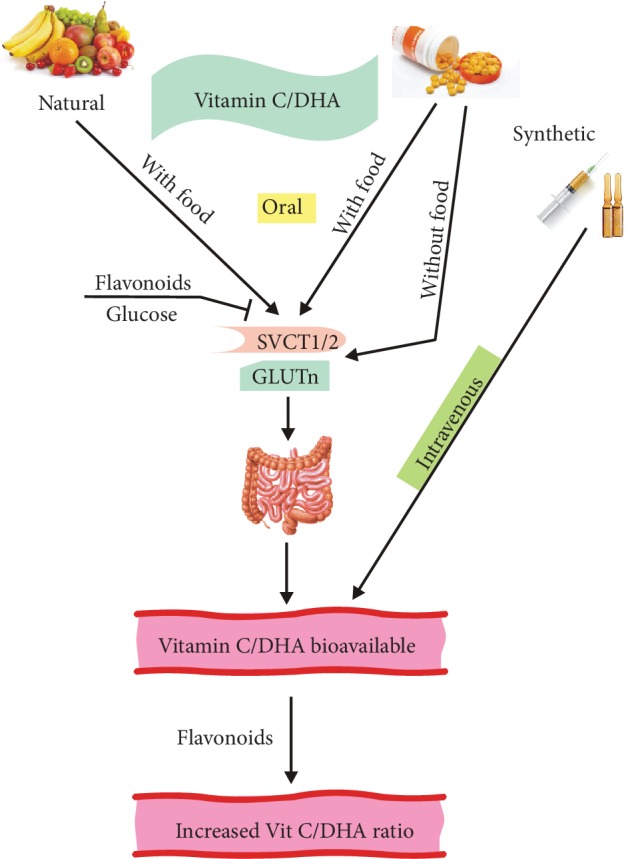
Absorption and bioavailability of natural and synthetic vitamin C. Vitamin C that is partly oxidized to dehydroascorbate (DHA) in an oxygen environment is transported by two sodium-dependent transporters SVCT1 and SVCT2, while DHA is taken up by the glucose transporter GLUT*n*, where *n* is 1-3 or 8. Vitamin C/DHA can be taken as either natural or synthetic ascorbic acid, and the latter can be given orally (with or without food) or intravenously. The final concentration of vitamin C in circulation depends not only on the route of ingestion but also on its excretion (not presented here) and the action of other dietary compounds, including glucose and flavonoids. Flavonoids can block the absorption of vitamin C, but they can also reduce some oxidants leading to an increase in the vitamin C/DHA ratio.

In general, vitamin C may be administered in three different ways—with food, as a food supplement, and as a synthetic product given independently of food, usually orally or intravenously (Figure 2). Chemically, natural and synthetic ascorbic acids are identical, but it is often stated that vitamin C from natural sources is better absorbed than its synthetic counterpart or the biological activity of natural vitamin C is superior to its synthetic formulations. These opinions are not supported by clinical studies that showed similar bioavailability of ascorbic acid from different natural sources, including oranges, broccoli, and kiwifruits, to that of synthetic vitamin C [15–18]. Moreover, the content of vitamin C as well as many other health-beneficial nutrients in fruits and vegetables decreases in time as shown by Davis et al. in their landmark publication indicating about a 60 percent decrease in vitamin C content in crops in 1999 as compared with 1950 [19].

Doses of vitamin C up to 2000 mg/day are considered safe for general consumption, but even so high doses are unlikely to result in plasma concentrations higher than 80 *μ*M [20]. Concentration of vitamin C in plasma is under control and is around 50 *μ*M, but intravenous administration of ascorbic acid may lead to transient, many-fold increase in that value [21, 22].

In a pharmacokinetic study, Levine et al. showed that the concentration of vitamin C in humans is under a tight control resulting from a coordinated action of multiple mechanisms and reached a saturation plateau of about 80 *μ*M after oral intake higher than 250 mg/day [23]. Moreover, these studies suggest that single oral doses higher than 200 mg are characterized by relatively low bioavailability, suggesting that such high dose should be rather divided into several subdoses [23]. When vitamin C is delivered intravenously, several elements of that tight control can be bypassed—the same amount of vitamin C administered intravenously may result in its six times higher concentration than when that amount is taken orally [23, 24]. Such a dramatic difference in actual concentration of vitamin C may explain differences in several cohort studies in which vitamin C pharmacokinetics was overlooked [25].

There are many problems with a reliable comparison of the bioavailability of natural and synthetic vitamin C. The proper experimental design in clinical trials requires an adequately large cohort to compensate for interindividual differences in metabolism of chemicals, digestion, and other aspects of biotransformation of ascorbic acid from different sources [18]. A few studies cited above were performed on relatively small populations with low statistical power. However, when the bioavailability of a chemically pure substance is compared with that of its natural counterpart, the latter usually acts in the context of other substances that may interfere with its action. Therefore, vitamin C ingested with natural products should be always considered along with other substances that may act synergistically with the vitamin, enhancing or decreasing its bioavailability and health-beneficial effects. Therefore, when vitamin C from a natural product is extracted, intermediate products of that extraction can induce decreasing bioavailability of the vitamin in plasma and lower profitable effects, although the nominal content of ascorbic acid in all these intermediates is the same [26]. This was supported by Vissers et al. who showed that kiwifruit provided a higher level of ascorbate in vitamin C-deficient mice than synthetic vitamin C [27]. However, the presence of natural components may also decrease bioavailability of vitamin C. As mentioned above, the oxidized form of ascorbic acid, DHA, can be transported by glucose transporters, but DHA must compete with glucose, which can be provided by many compounds present in natural sources of vitamin C. Moreover, some flavonoids, plant-derived substances of general health-beneficial influences, were reported to inhibit vitamin C and DHA transporters both *in vitro* and *in vivo* [12, 28–30]. On the other hand, flavonoids display antioxidant properties and their action can spare molecules of vitamin C; otherwise, they are oxidized [31].

Not only natural vitamin C from different sources but also its synthetic counterpart displays different bioavailability [32, 33]. Bioavailability of vitamin C is determined not only by its uptake but also by its renal excretion. Among many formulations of synthetic vitamin C, the highest bioavailability potential has slowly releasing compounds and salts of vitamin C, at least in animal studies [34, 35].

Flavonoids may modulate bioavailability of vitamin C. Animal studies with flavonoid-rich extracts or purified plant flavonoids showed an enhanced uptake of vitamin C when it was administered together with flavonoids [36, 37]. These results were supported by studies on scorbutic guinea pig showing a decrease in the number of hemorrhages in animals receiving vitamin C with quercetin or rutin as compared with vitamin C singly [38]. On the other hand, both *in vitro* and *in vivo* studies suggest that certain flavonoids can inhibit the uptake of vitamin C and DHA by inhibiting their transporters [30]. However, the studies showed that the influence of flavonoids on the bioavailability of vitamin C is limited by their low plasma concentration [39]. Moreover, Lotito and Frei showed that increased plasma antioxidant capacity was not induced by flavonoids derived from apple consumption but resulted from metabolic effects of fructose on urate [40]. Therefore, the effect of flavonoids on bioavailability of vitamin C in human is not completely known and it likely depends on cellular metabolic status, but several works suggest that it may be of marginal significance [15].

Animal studies suggest a higher bioavailability of natural than synthetic vitamin C, but all human studies do not indicate such a difference [41]. It may provoke a question about research design in both kinds of studies. Animals can be studied in a more reliable way due to a more exact match of studied and control groups, strictly controlled diet and environmental condition, and lesser ethical limitations allowing obtaining tissues and organ not accessible in humans. Difference in bioavailability of natural and synthetic vitamin C in animal studies and a general lack of such a difference in human studies suggest a need for more rigorous trials.

Synthetic vitamin C is reported to enhance bioavailability of health-beneficial nutrients, including vitamin E and nonheme iron that may increase health effects of foods that contain vitamin C [42, 43].

To determine anticancer potential of vitamin C, it is important to determine the difference between its bioavailability in normal and cancer cells, especially that the results of studies suggest that such a difference can depend on the type of cancer [44–47]. Given that vitamin C transporters SVCT1 and SVCT2 are essential in the acquisition of this vitamin by the cell, Pena et al. showed that breast cancer samples differentially expressed a form of the SVCT2 transporter, systematically absent in normal breast tissue [48]. However, these authors observed that various cancer cell lines were not able to uptake vitamin C and acquired it by a glucose transporter by a bystander effect. Furthermore, that specific form of SVCT2 was absent in the plasma membrane, but it was overexpressed in mitochondria of cancer cells. Therefore, cancer cells may uptake vitamin C in its oxidized form (DHA) and accumulate high concentrations of its reduced form.

## 3. Human Studies

This is not the main subject of this review to present results of human studies on vitamin C supplementation in cancer, because in many cases, they suffer from many methodological drawbacks. Only some studies are presented.

In a large cohort study in France—the Etude Epidémiologique aupre`s de femmes de la Mutuelle Générale de l'Education Nationale (E3N)—the association between invasive breast cancer and vitamin C intake was analyzed in 2482 cases [49]. Data on vitamin C ingestion, both from supplementation and natural products, were obtained from validated food frequency ever-never-type questionnaires covering a few years' periods. Ever use of vitamin C in the form of supplements resulted in a nonsignificant pooled OR.

In a meta-analysis of 37 studies, it was concluded that the total (dietary and supplementary) intake of vitamin C reduced breast cancer risk by 15%, but dietary-only intake of the vitamin reduced that risk by 23% [50]. However, results on supplementation with vitamin C suggested a higher risk of breast cancer, but this relationship was not relevant as pooled OR became nonsignificant in all but one case.

High vitamin C intake from food was associated with a decreased risk of breast cancer in a case-control study, but such association was not observed in prospective epidemiological studies [50].

A weak positive association between dietary and supplementary vitamin C use and breast cancer was observed in 2879 cases of invasive breast cancer in the study within the Women's Health Initiative Observational Study [51]. Women enrolled in that study consumed daily on average 106 mg vitamin C from the diet, and about 60% of them took supplemental vitamin C on an average dose 350 mg per day. There was also an association between breast cancer occurrence and the highest (>686 mg per day) quintile of total vitamin C intake as compared with its lowest (<97 mg per day) counterpart. No association between dietary vitamin and breast cancer was observed. These studies also brought some suggestions on association between vitamin C intake and occurrence of hormone receptor status-specific types of breast cancer, but those analyses were performed on much smaller populations than the general study population.

Many studies, including a randomized clinical trial with 10-year supplementation with 500 mg vitamin C per day, reported no association between either dietary or supplementary vitamin C intake and breast cancer [52–55].

A recent update of literature on the influence of vitamin C on prostate cancer shows many nonconclusive findings but in general concludes that dietary intake of vitamin C and other elements of healthy diet is promising in the prevention and therapy of prostate cancer [56]. However, “promising” is elusive.

An initial report on anticancer action of high (pharmacological) doses of vitamin C was provided by Benade et al. in 1969, although the first evidence of this effect was documented in the 30s of the last century [57]. Then, Cameron and Pauling observed a more than 4 times extension of survival in patients in the terminal state in various cancers who received high concentrations of intravenously administered ascorbic acid as compared with similar patients who did not receive such treatment [58]. The rationale to undertake the research was that cancer patients were characterized by an apparent lack of ascorbic acid whose metabolism was involved in a number of natural anticancer mechanisms [59]. Although it was postulated that vitamin C might selectively act as a prooxidant in cancer cells, these studies needed explanation on the molecular basis.

Han et al. proposed another mechanism of anticancer action of vitamin C [60]. They observed that the levels of this vitamin were correlated with the mRNA expression of the transmembrane protein with epidermal growth factor- (EGF-) like and two follistatin-like domains 2 (TMEFF2) in gastric cancer (GC) patients. As TMEFF2 is downregulated in GC and correlated with tumor aggressiveness, so restoring the physiological level of vitamin C in GC patients may limit the progression of this malignancy, and conceptually this can be reached by the dietary supplementation. This is in line with an inverse relationship between dietary vitamin C intake and GC occurrence observed in a Korean cohort study [61].

Guarnieri et al. compared the influence of a single portion of natural vs. synthetic vitamin C on DNA damage induced by hydrogen peroxide [62]. They observed similar plasma concentrations of this vitamin in 7 volunteers, but only natural vitamin, consumed from orange juice, decreased the extent of H_2_O_2_-induced DNA damage in peripheral blood mononuclear cells. The authors concluded that this might not be vitamin C itself that was directly responsible for the protective effects against DNA damage, but rather other compounds, like phytochemicals that might synergize the action of the vitamin or act independently of it.

## 4. Molecular Studies

Park et al. showed that vitamin C induced phosphorylation of extracellular signal-regulated kinases (ERK) and resulted in the activation of its catalytic domain in AML (acute myeloid leukemia) cells [63]. They also showed that the small G-proteins Raf1 and MAPK-activated protein kinase 2, an upstream and a downstream regulator of ERK, respectively, were induced by the vitamin. The minimal concentration of vitamin C in those experiments was 100 *μ*M, and it induced a significant effect, so it can be speculated that these effects could be induced by dietary vitamin C. Later studies of these authors showed that vitamin C at high concentrations was beneficial with no adverse effects for patients with AML or myelodysplastic syndromes (MDS) [64]. This was confirmed by Mastrangelo et al. who showed that high concentrations of sodium ascorbate—0.5-7.0 mM—were cytotoxic for many myeloid-derived cancer cell lines contrary to normal cells derived from human cord blood [65].

Bhat et al. showed that ascorbic acid at the concentrations from the range 100-200 *μ*M induced oxidative DNA damage in normal human peripheral blood lymphocytes [66]. The damage was ameliorated by the sequestering of copper ions (Cu(I)) suggesting that Cu(I) is an intermediate in DNA-damaging action of ascorbic acid. These results show that an antioxidant, which is considered to be ascorbic acid, may act as a prooxidant in specific conditions, including copper overload. Furthermore, this effect may underline the anticancer action of high concentrations of ascorbic acid observed in early experiments of Pauling [67]. This hypothesis is supported by studies reporting a higher concentration of copper in cancer [68–70]. Many cancers are characterized not only by increased intratumoral concentrations of copper but also by altered systemic distribution of that element (reviewed in [71]). Copper is needed in cancer cells to keep a rapid proliferation, as it is a cofactor of enzymes involved in DNA replication. It also plays a role in cancer progression.

Chen et al. demonstrated that intravenous administration of ascorbic acid at high concentrations was toxic for many types of cancer cells in xenografts in mice with no effect on normal cells [72]. The authors suggested that ascorbic acid could support the formation of hydrogen peroxide in cancer cells leading to oxidative stress and cell death. However, it was not completely clear why normal cells were resistant to such cytotoxic action of vitamin C. To address this problem, Ullah et al. showed that ascorbic acid mobilized copper from the nuclei of human peripheral blood lymphocytes [73]. That copper was involved in redox cycling by ascorbic acid or extracellular ROS and contributed to DNA damage in cancer cells. As cancer cells contain more copper than their normal counterparts, they are more prone to electron transfer between copper ions and ascorbic acid to generate ROS. Moreover, cancer cells may have impaired the antioxidant system as they need a high level of ROS to proliferate and promote other effects implicated in tumor growth and progression [74].

Rawal et al. showed that catalytic manganoporphyrins increased the ability of ascorbate to donate electron to molecular oxygen to generate hydrogen peroxide and enhance its toxicity in cancer cells [75]. Schoenfeld et al. showed that glioblastoma and non-small-cell lung cancer cells are selectively sensitive to ascorbate due to their changed redox-active iron metabolism [76].

**Figure 3:**
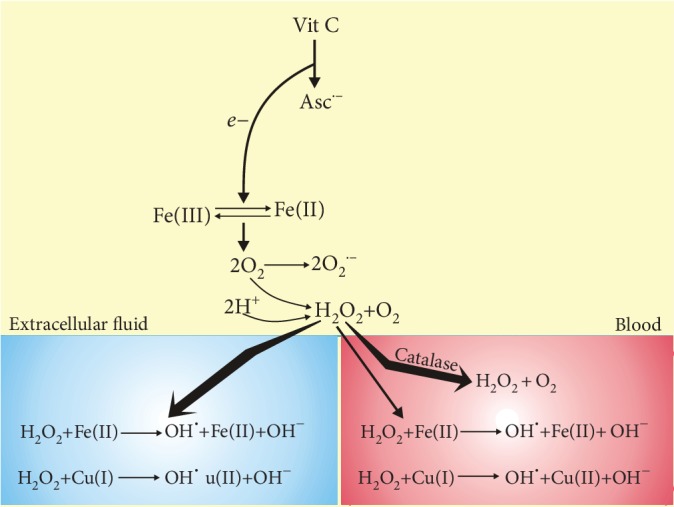
Vitamin C may differently produce reactive oxygen species (ROS) in blood and the extracellular space. After oral or intravenous administration, vitamin C reaches the same concentration in blood and extracellular fluid and loses one electron (*e*^−^) to form ascorbate radical Asc^·−^ and reduces a protein-centered metal ion, such as Fe(III). Reduced metal donates an electron to oxygen forming ROS, including superoxide (O_2_^·−^) that can be dismutated to hydrogen peroxide. These reactions in blood are inhibited by plasma and red cell membrane proteins, and hydrogen peroxide in blood is neutralized by antioxidant enzymes hardly present in extracellular fluid. Unless H_2_O_2_ is decomposed, it may produce hydroxyl radicals in the Fe(II)- or Cu(I)-catalyzed Fenton-like reaction yielding hydroxyl peroxide (HO^·^).

Ascorbate may reduce intracellular ferric (Fe(III)) ions to ferrous (Fe(II)) ions that can react with oxygen to produce the superoxide anion—one such anion is produced for each molecule of ascorbate interacting with iron [77]. Superoxide, in turn, can be involved in the production of hydrogen peroxide, which can be decomposed to produce hydroxyl and other radicals. Chen et al. pointed at the fact that this chain of reactions would preferentially occur in the extracellular space than in blood where it would be inhibited by plasma and red cell membrane proteins [78] (Figure 3). Moreover, hydrogen peroxide in blood is decomposed by antioxidant enzymes, primarily catalase. Hydrogen peroxide can produce the hydroxyl radical in the Fe(II)- or Cu(I)-dependent Fenton-like reaction. As mentioned, some malignant tumors are rich in Cu(I), so HO^·^ could be preferentially produced in cancer tissue. Moreover, as cancer cells have a reduced activity of antioxidant enzymes, they can be selectively killed by the action of free radicals [79]. Other transition metals can also catalyze the production of hydroxyl radicals. Does dietary vitamin C have something to do with that effect? In practice, it requires high concentrations of vitamin C due to relatively low level of transition metals. Such high concentrations of the vitamin could not be achieved by oral administration.

This chain of reactions is supported by the results of many *in vitro* studies showing selective killing of cultured cancer cells by ROS as they often display deficient antioxidant defense. However, the presence of iron determines ROS production and its concentration can matter for the final outcomes. This is especially important as most of the *in vitro* studies on anticancer action of vitamin C are performed in culture media that are poor in iron as compared with the plasma. However, in most studies, concentration of iron was neither measured nor taken into account. This problem was addressed by Mojic et al. who showed that the cytotoxic effect of vitamin C in LNCaP and PC-3 prostate cancer cell lines as well as in primary astrocytes was abolished by iron at physiological concentrations [80]. At low iron concentration, ascorbate reduces Fe(III) to Fe(II) that reacts with molecular oxygen and produces superoxide that can be dismutated to hydrogen peroxide, which can penetrate the cell membrane and cytoplasm. At high iron concentrations, H_2_O_2_ will be decomposed before entering the cell and oxidate ascorbate and proteins. This important study points at the possibility of inducing/enhancing anticancer effects of vitamin C *in vivo* by iron chelating. However, at present, it is rather a complex issue as many questions must be answered, including an optimal and still physiologically relevant iron concentration to produce free radicals and decompose hydrogen peroxide. That study was supported by the recent work of Tsuma-Kaneko et al. who showed that excess of iron decreased the pharmacological vitamin C-induced inhibition of survival of the human myeloid leukemia K562 cells *in vitro* through a decrease in H_2_O_2_ level and depletion in the apoptotic pathway induced by the vitamin [81]. Moreover, iron excess reversed anticancer effects induced by vitamin C *in vivo*, so stimulation, not inhibition, of the K562 cell in mouse transplants was observed. These data strengthen the need for close control of iron concentration in experiments on anticancer action of vitamin C and possible reinterpretation of some previous studies. These and other studies show that ascorbate toxicity *in vitro* depends on the kind of culture media and cannot always determine its toxicity *in vivo* as iron that is critical for ascorbate-induced effects is usually sequestered in transferrin and ferritin in plasma and mostly redox-inactive [82, 83].

All these experiments suggest that when the concentration of catalytic metal ions is high enough to support electron transfer between this element and ascorbate, hydroxyl radicals and other radicals can be formed to damage DNA [84]. This creates a potentially lethal state for a cell that contains elevated level of copper and is influenced by high concentrations of ascorbate. However, DNA damage in cancer cells increases the level of genomic instability, typical of the most if not all cancer cells, that may increase advantages of these cells over their normal counterparts and result in tumor growth and progression. Therefore, these anticancer effects of high concentrations of vitamin C can have several implications for copper-oriented anticancer therapy.

Recently, Graczyk-Jarzynka et al. showed that malignant B cells used the thioredoxin antioxidant system to scavenge hydrogen peroxide that had been generated outside a cell [85]. Inhibition of peroxiredoxin 1, an H_2_O_2_-removing enzyme, increased the sensitivity of malignant B cells to vitamin C. Moreover, auranofin, a thioredoxin inhibitor, decreased H_2_O_2_ scavenging in these cells, suggesting that it might act synergistically with vitamin C, which was confirmed in further experiments. Therefore, a new mechanism of anticancer action of vitamin C is proposed along with the suggestion of a combined anticancer therapy with this vitamin and auranofin.

Cu(II) may lead to autoxidation of vitamin C, but some flavonoids, including quercetin, morin, and catechin exerted a protective effect *in vitro* against such outcome [31]. This reaction can be mainly underlined by a strong scavenger activity towards ROS and reactive nitrogen species, chelating metals involved in free radical generation and activation of antioxidant enzymes (reviewed in [86]).

**Figure 4:**
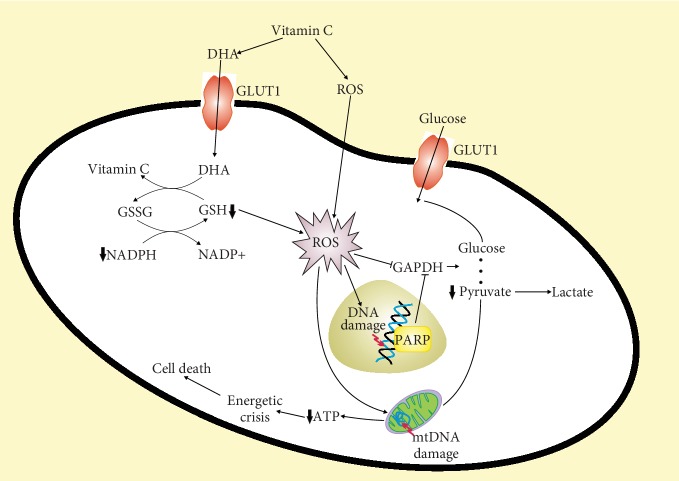
Vitamin C is oxidized in the extracellular space to dehydroascorbate (DHA) that is taken up by cancer cells via glucose transporters such as GLUT1. Inside the cell, DHA is reduced back to vitamin C by reduced glutathione (GSH) that is oxidized to glutathione disulfide (GSSG) and converted back to GSH by reduced nicotinamide adenine dinucleotide phosphate (NADPH). Depletion of GSH and NADPH results in ROS overproduction that may damage biomolecules and kill cancer cells. ROS-damaged DNA activates poly(ADP-ribose) polymerase (PARP) that requires NAD+. ROS can also inhibit glyceraldehyde 3-phosphate dehydrogenase (GAPDH) resulting in decreased production of pyruvate and ATP by mitochondria and finally energetic crisis and cell death [82–84].

Yun et al. showed that human colorectal cancer cells (CRC) harboring mutations in either the *KRAS* or *BRAF* gene were selectively killed by vitamin C at high concentrations [87]. Moreover, vitamin C inhibited tumor growth in mice bearing the G12D mutation in the *KRAS* gene. These effects were attributed to the inactivation of glyceraldehyde 3-phosphate dehydrogenase (GAPDH) by ROS that are nor effectively scavenged due to depletion of glutathione. Glutathione was depleted by oxidative stress caused by increased DHA uptake via the GLUT1 glucose transporter and reduction of DHA back to vitamin C. Colorectal cancer cells with *KRAS* or *BRAF* mutation depend more on glycolysis than their nonmutated counterparts, not to mention normal cells. Inhibition of glucose transport in these cells due to competition between glucose and DHA for glucose transporters may lead to decreased ATP production, energetic crisis, and eventually cell death. It was also found that ROS evoked by high doses of vitamin C induced DNA damage that activated PARP, which in turn causes NAD^+^ depletion resulting in the inhibition of glycolysis. This important work suggests that the oxidized form of vitamin C, DHA, is its pharmaceutically active agent and that high expression of the GLUT1 transporter in cancer cells combined with mutations causing glycolytic addiction may be responsible for a selective anticancer effect of vitamin C (Figure 4). Although these studies were performed on cells with specific mutations, anticancer effect of vitamin C was underlined by switching on glycolysis that is typical of most of cancers. However, these studies were carried out on cell cultures and transgenic mice, and it is an open question how they can be translated into humans. So high-millimolar concentrations of vitamin C in circulation can be reached only intravenously, but not with its oral administration, even in the form of tablets or liquid [22].

These results suggest that the metabolomic profile of cancer cells can be important in cancer-related effects of vitamin C. This issue was addressed by Uetaki et al. who showed that vitamin C at high concentrations changed the profile in human breast and colon cancer cell lines [88]. These changes included an increase in the levels of upstream metabolites of the glycolysis pathway and tricarboxylic cycle as well as a decrease in adenosine triphosphate (ATP) levels and adenylate energy charges. Changes in the metabolic profile induced by vitamin C are associated with energy depletion underlined by NAD deficiency and may ultimately lead to cancer cell death.

Hydrogen peroxide-mediated anticancer effect of vitamin C was confirmed by Rouleau et al. who showed that this vitamin acted synergistically with the chemotherapeutic sorafenib in killing the hepatocellular carcinoma (HCC) Hep G2 cells [89]. They indirectly showed that high concentrations of vitamin C (5-20 mM) selectively produced H_2_O_2_ in cancer cells as compared with their normal counterparts. Moreover, a lower concentration of this vitamin (1 mM) enhanced cytotoxic effects of H_2_O_2_-generating glucose oxidase. Vitamin C deregulated calcium homeostasis resulting in calcium accumulation in mitochondria, and sorafenib induced mitochondrial depolarization and prevented calcium sequestration in mitochondria. These *in vitro* studies were supported by the case of an HCC patient who displayed regression of metastasis after combined treatment with vitamin C and sorafenib.

Vitamin C was shown to enhance the cytotoxic action against cancer cells *in vitro* and in mouse xenograft models exerted by auranofin, a redox-modulating drug targeting simultaneously thioredoxin and glutathione antioxidant systems [90]. Such combined action was efficient in triple-negative breast cancer and linked with the expression of prostaglandin reductase 1 (PTGR1).

Vitamin C increased the efficacy of DNA double-strand break (DSB) induction in 2D human lung cancer cell cultures and 3D spheres by bleomycin, an anticancer drug [91]. Vitamin C was effective also when low concentrations of bleomycin were combined with inhibitors of ATM (ataxia telangiectasia mutated) and the catalytic subunit of DNA-dependent protein kinase (DNA-PKcs) that are proteins essential in DSB repair.

## 5. The Immune System

The immune system is primarily responsible for both prevention of and fighting with cancer. Beneficial effects of vitamin C for the immune system are commonly known (reviewed in [92]). On the other hand, cytotoxic innate and adaptive immune cells are a destructive barrier for cancer progression, including metastasis [93]. Therefore, studies on the role of vitamin C in the activity of the immune system in cancer are justified and have been recently reviewed by Ang et al. [3].

Normal concentration of vitamin C in immune cells is in a millimolar range resulting from its plasma concentration about 50 *μ*M corresponding to an intake of 100 mg daily by a healthy individual [23].

Growth of solid tumors is associated with hypoxia, so tumor cells must have tools to survive in hypoxic conditions. One of such tools is hypoxia-inducible factor 1 (HIF-1) consisting of two subunits HIF-1*α* and HIF-1*β* that regulate the expression of genes implicated in metabolic reprogramming with the involvement of GLUT1, angiogenesis, antiapoptotic mechanisms, stem cell renewal, invasion, and metastasis as well as therapeutic resistance of cancer cells [94, 95]. Hydroxylation of HIF-1*α* in normoxia induces its proteasomal degradation, but hypoxia inhibits that process leading to increased stability and transcriptional activity of HIF-1*α*. Such hydroxylation requires vitamin C for optimal activity of the hydroxylase enzymes [96]. Many aspects of the interaction of vitamin C with the immune system relate to HIF-1/2 [3].

Monocytes display a high intrinsic concentration of vitamin C that can be related to HIF dependency on their functionality [23]. Activation of HIF-1/2 in monocytes in cancer led to the induction and development of tumor-associated macrophages that are linked with the expression of immunosuppressive and protumor proteins that leads to an increased tumor invasion and suppression of T cells; otherwise, it is toxic for cancer cells [97, 98].

Cancer cells are characterized by an increased resistance to apoptosis, which is the main kind of cancer cell death induced by anticancer therapy. Pharmacologic concentrations of vitamin C in fresh human monocytes and a monocytic cell line were associated with inhibition of Fas-induced apoptosis, reduction of the activity of caspase-3, caspase-8, and caspase-10, reduced ROS levels, and increased permeability of the mitochondrial membrane [99].

NK cells isolated from *Gulo^−/−^* mice that are a model of human ascorbate dependency condition, whose diet was deprived of vitamin C for 2 weeks, displayed a decreased *in vitro* killing efficacy against ovarian cancer cells as compared with those from animals receiving full supplementation with vitamin C [100]. These cells secreted less interferon gamma (IFN-*γ*) after coculturing with cancer cells and showed a decreased expression of perforin and granzyme B. *Gulo^−/−^* mice showed a shorter survival time than control animals. It was concluded that normal plasma concentration of vitamin C is essential for NK stimulation against cancer cells. Therefore, natural NK activity, declined in cancer, can be restored and maintained by dietary supplementation of vitamin C.

NK cell stimulation by vitamin C can be important in the reconstitution of the immune system after immunosuppression as it occurs after myeloablative chemotherapy or allogeneic hematopoietic cancer stem cell transplantation (HCSCT) in leukemias as NK cell reconstitution is faster than that of their T counterparts [101]. In this way, NK cells can provide a transient immunity to infections. However, some studies suggest that vitamin C supplementation leading to increased amount and function of regulatory T lymphocytes can decrease the graft against tumor effect occurring after HCSCT [102].

Antioxidants, including vitamin C, induce HIF-1*α* degradation that may underline anticancer effects of this vitamin [103]. Rouleau et al. demonstrated that vitamin C and its oxidation-resistant derivative, ascorbate-2-phosphate (A2P), downregulated HIF-1*α* in melanoma cells reducing their invasive potential [89]. Fischer and Miles showed that vitamin C and A2P downregulated HIF-1 in melanoma cells and that this effect was associated with a decrease in malignant properties of those cells, including their invasiveness [104]. These authors did not observe such effect for the treatment with DHA. Kuiper et al. showed that the concentration of ascorbate decreased in low-grade endometrial tumors and was associated with a high HIF-1*α* expression and an increased tumor size [46]. In their subsequent work, these authors observed that high levels of ascorbate in colorectal tumors were associated with low levels of HIF-1*α* and lower expression of its downstream products [45]. However, the authors could observe only a correlation between ascorbate concentration and HIF-1*α* expression and other quantities they investigated as the level of ascorbate could not be manipulated. To overcome this limitation, Campbell et al. employed C57BL/6 Gulo^−/−^ mice whose diet was supplemented with 33, 330, or 3300 mg/L of ascorbate before and during subcutaneous tumor growth of melanoma or lung carcinoma [105]. Both tumors were characterized by a decreased level of ascorbate. A reduction of tumor growth was observed with increased intake of ascorbate leading to restoration of its optimal intracellular level. These results support the hypothesis that ascorbate is required for the regulation of HIF-1 and such regulation is negative in tumor tissue. Low (33 mg/L) dose of ascorbate also reduced tumor growth suggesting that restoring the optimal level of ascorbate to inhibit tumor growth can be reached by dietary vitamin C.

Antitumorigenic effect of vitamin C mediated by lowering HIF-1 activity was confirmed by Gao et al. who showed that this vitamin inhibited a MYC-dependent human B lymphoma model in a prolyl hydroxylase 2 and von Hippel-Lindau protein-dependent manner [103]. Many studies suggest that HIF-1 can be targeted by vitamin C to exert its anticancer effects, but not all tumors are HIF-1-dependent [106].

Many functions of vitamin C in the mobilization of the immune system against cancer relate to its regulation of the epigenetic profile of immune cells.

## 6. Epigenetic Profile

Epigenetic modifications are changes in the genome that are not directly related to changes in the DNA sequence. These modifications may change the gene expression pattern and include DNA methylation/demethylation, posttranslational modifications of histones, and consequences of the action of noncoding RNAs. DNA methylation is carried out by DNA methyltransferases or the direct action of methylating agents, and its demethylation occurs spontaneously, passively or is catalyzed by ten-eleven translocations (TETs) (actively).

Vitamin C was shown to enhance anticancer action of two epigenetic drugs decitabine and azacytidine in CRC cells [107]. These drugs demethylate DNA, and administration of vitamin C with either of them increased the level of 5-hydroxymethyl-2′-deoxycytidine (5-hmdC). This effect was associated with an increased expression of the p21 tumor suppressor and induction of apoptosis. These results suggest that vitamin C can support anticancer therapy based on demethylating drugs. Significant effects were observed from 10 *μ*M vitamin C in the cell, so a dietary supplementation of this vitamin can be considered to support the action of epigenetic drugs in colorectal cancer.

Clear cell renal cell carcinoma (ccRCC) is associated with aberrant hypermethylation of cytosine in DNA [108]. Shenoy et al. showed that loss of 5-hydroxymethylcytoine (5hmC) was associated with a more aggressive phenotype of ccRCC suggesting that this effect could be considered a molecular diagnostic or prognostic marker of ccRCC and potential therapeutic target [109]. Searching for the mechanism underlying the observed effects, the authors noted a functional inactivation of TETs by L-2-hydroxyglutarate (L2HG) that was overexpressed due to deletion and resulting underexpression of the *L2HG* dehydrogenase gene. Vitamin C at 0.1 and 1 mM decreased DNA methylation and activated TET restoring genome-wide 5hmC levels. The increased level of intratumoral 5hmC induced by high doses of vitamin C was associated with reduced tumor growth. However, interaction of the recombinant TET2 protein with vitamin C was also observed at relatively low concentrations of this vitamin (10-40 *μ*M), but it is not known whether that interaction is clinically relevant. This work indicates another mechanism of anticancer effect of vitamin C that is not related to its antioxidant action. The mechanism of stimulation of TET by vitamin C was proposed as a direct interaction of the vitamin with the TET catalytic center with concomitant promotion of TET folding resulting in the improvement of Fe (II) recycling [110]. Alternatively, vitamin C may act as a cofactor of TETs stimulating their activity by the reduction of Fe (III) to Fe (II) [111].

Other aspects of the interaction between vitamin C and TET important for cancer stem cells are presented in the next section.

## 7. Cancer Stem Cells

Malignant tumors are characterized by intrinsic heterogeneity displayed in a diverse array of structurally and functionally different cells. A small proportion of the cells have been identified as primary responsible for disease recurrence and resistance to therapy. This led to the conception of cancer stem or cancer stem-like cells that are the ultimate target in cancer therapy [112]. Induced pluripotent stem cells (iPSCs) have opened a new perspective in modeling human cancers and anticancer cellular and gene therapies [113]. Although not directly related to the subject of this review, it is worth mentioning that vitamin C was reported to facilitate generation of iPSCs by a defined factor by the epigenetic-related mechanisms with the upregulation of histone deacetylates Jhdm 1a/b (Jumonji C domain-containing histone demethylase 1A), JARID1A (Jumonji, AT rich interactive domain 1A), and JMJD3 (Jumonji domain-containing protein 3) as well as the TET proteins [114–117].

Combined treatment of CSCs with doxycycline and vitamin C resulted in eradication of CSCs originating from the MCF7 breast cancer cells; otherwise, they are resistant to doxycycline [118]. It was argued that the observed effect was due to synthetic lethality induced by the combined treatment in that doxycycline targeted mitochondrial respiration and vitamin C—glycolysis. The effectiveness of the combined treatment with doxycycline and vitamin C in cancer by targeting mitochondria was confirmed in subsequent studies in which azithromycin was also applied [119].

Using a metabolomics method, Agathocleous et al. showed that human and mouse hematopoietic stem cells (HSCs) had unusually high levels of ascorbate that decreased with differentiation [120]. Systemic reduction of vitamin C in mice deficient in ascorbate synthesis (*Gulo^−/−^*) increased the fraction and functions of HSCs underlined, at least in part, by reduced function of the TET2 protein. Mutations inactivating the *TET2* gene belong to the earliest molecular events in leukaemogenesis in humans, increasing HSCs and self-renewal [121]. Therefore, vitamin C accumulates in HSCs and activates the TET proteins resulting in a decrease in HSC fraction and suppression of leukaemogenesis.

**Figure 5:**
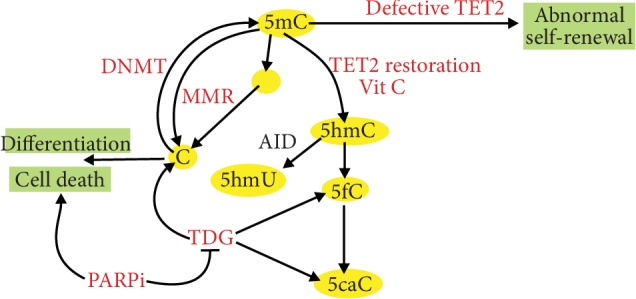
Vitamin C induces the ten-eleven translocation 2 (TET2) proteins to kill leukemic blasts. TETs are involved in active DNA demethylation that is achieved through TET2-mediated oxidation of 5-methylcytosine (5mC) to 5-hydroxymethylcytosine (5hmC), 5-formylcytosine (5fC), and 5-carboxylcytosine (5caC). Oxidized 5mC is progressively lost in subsequent cellular divisions or converted to nonmethylated C by thymine DNA glycosylase (TDG). 5mC can undergo spontaneous or activation-induced deaminase- (AID-) mediated deamination converting it into thymine (T) that can be replaced by C by TDG or in mismatch repair (MMR). AID can convert 5hmC to 5-hydroxymethyluracil (5hmU) or T. If TET2 is deficient in leukemic stem cells, their self-renewal is disturbed leading to increased blast production and progression of the disease. Vitamin C exerts similar effects as restoration of TET2 that leads to increased differentiation and less aggressive disease. Vitamin C-induced oxidation of 5mC results in an increased sensitivity of the cells to inhibitors of poly(ADP-ribose) polymerase (PARPi) that can induce cell death and inhibit disease progression.

Using reversible RNAi mouse and cell cultures, Cimmino et al. showed that TET2 deficiency resulting in aberrant self-renewal of HSCs and progenitor cells *in vivo* and *in vitro* was reverted by TET2 restoration [122]. Such effect was also obtained by the treatment of HSCs and their progenitors obtained from TET2-deficient mice with vitamin C that increased the formation of 5-hydroxymethylcytosine (Figure 5). Vitamin C also suppressed the colony formation of human leukemic cells and progression of human primary leukemia patient-derived xenografts. Vitamin C induced DNA hypomethylation and the expression of the TET2-dependent genes. Vitamin C treatment induced TET-mediated DNA oxidation in leukemic cells making them more sensitive to poly(ADP-ribose) polymerase (PARP) inhibition. The authors typically used vitamin C at 250 *μ*M, but in several experiments, they observed a pronounced effect at 125 *μ*M, so its interpolation allows speculating that vitamin C would exert a relevant antileukemic effect at concentrations that can be reached by dietary supplementation.

5-Methylcytosine may undergo induced or spontaneous deamination resulting in its conversion to thymine and formation of the G:T pair. This mispairing is targeted by thymine DNA glycosylase (TDG) of the base excision repair or mismatch repair (MMR) system that may restore cytosine in the place of thymine. Catani et al. showed that vitamin C upregulated the MLH1 (Mut L homologue-1) protein in human keratinocytes [123]. The cells supplemented with vitamin C showed greater sensitivity to apoptosis induced by cisplatin underlined by the activation of the MLH1/c-Abl/p73 signaling pathway. Apart from a perspective to use vitamin C to increase the efficacy of anticancer drugs, this work contributes to another role of this vitamin—to keep genomic stability as MMR is a principal and in practice the only one mechanism dealing with replication errors that were not corrected by DNA polymerase. As genomic instability is an essential feature of cancer transformation, vitamin C may be involved in basic mechanisms of carcinogenesis.

Kim et al. showed that high, up to 5 mM, concentrations of vitamin C inhibited the formation of spheres, typical of CSCs, in neural stem/progenitor cells (NSPCs) and upregulated some apoptotic genes underlined by a decrease in GSH and NAD^+^ [124]. Undifferentiated cells were more sensitive to the vitamin than their differentiated counterparts, likely due to increased expression of the genes encoding glucose transporters 1 and 3 (Glut1/3). Furthermore, vitamin C induced more DNA damage in the form of double-strand breaks in undifferentiated CSCs than differentiated cells. Therefore, vitamin C showed the potential to eradicate CSCs.

Ramezankhani et al. showed that vitamin C inhibited the expression of pluripotency factors, including OCT4A, SOX2, and NANOG in breast cancer cell lines with an overexpressed embryonic stem cell-specific miR-302/367 cluster that induces epigenetic reprogramming, which is known to induce tumor-suppressive functions in many cancers [125]. Moreover, treatment with the vitamin increased cancerogenic properties of breast cancer cells, including their invasiveness and resistance to apoptosis. These effects were associated with downregulated expression of the *TET1* gene. These results confirm that TET functions can play a role in anticancer strategies including reprogramming and vitamin C. The concentration of vitamin C in these experiments was 100 *μ*g/mL, nearly the range that can be achieved by dietary supplementation.

Opposing effects of low (5-25 *μ*M) and high (100-1000 *μ*M) concentrations of vitamin C were observed in cancer stem cells (CSCs) fractionated from the human colorectal cancer cell line HT-29 [126]. At low concentrations, vitamin C stimulated proliferation of CSCs, but it did not affect nonstem cancer cells and normal fibroblasts. At high vitamin C concentration, a decrease in proliferation of both CSCs and nonstem cancer cells and the effect were more pronounced in the latter two lines than in CSCs. That work did not clearly explain the reason of such differences, and further specific characterization of CSCs related to their interaction with vitamin C is needed.

## 8. Metastasis

Metastasis is responsible for most (approx. 90%) of cancer-related deaths [127]. This process requires adhesion to and invasion of surrounding tissues and organs by cancer cells and their movement through bloodstream to distant sites, where they initiate the formation of secondary tumors. Therefore, penetrating cellular and extracellular compartments by cancer cells is necessary for metastasis.

Collagen, a group of fibrous proteins fundamental in fibrils of the extracellular matrix (ECM) and the connective tissue, is a barrier in migration of cancer cells that must be broken in cancer invasion [128]. Therefore, cancer progression is associated with remodeling of ECM that is performed by a controlled degradation of ECM components with many proteins involved in this process, including matrix metalloproteinases (MMPs) that are a family of zinc-dependent endopeptidases [129]. As vitamin C promotes collagen synthesis, it can affect cancer growth, invasion, and metastasis [130].

Dietary vitamin C in combination with proline, lysine, and green tea extract was shown to inhibit invasion and metastasis as well as MMP secretion in many human cancer cell lines and animal models [131–135]. Vitamin C and its four derivatives, Asc-6-O-palmitate (Asc6Plm), Asc-2-O-phosphate (Asc2P), Asc-2-O-phosphate-6-O-palmitate (Asc2P6Plm), and Asc-5,6-benzylidene (Asc5,6Bz), inhibited the invasion of the human fibrosarcoma HT-1080 cells [136]. In addition, Asc2P6Plm administered intravenously inhibited pulmonary metastasis in mice injected with the B16BL6 melanoma cells. This derivative of vitamin C is more lipophilic than the parental compound, so its bioavailability could be higher, suggesting that synthetic lipophilic derivatives of vitamin C can display a more effective anticancer action than their natural counterparts. Although this is likely true for every lipophobic drug, these studies provide specific formulation and conditions of incubation that are important in an anticancer strategy with vitamin C.

Vitamin C, DHA, and D-isoascorbic acid inhibited the activity of testicular hyaluronidase and hyaluronan lyase, enzymes that are involved in the degradation of high molecular weight hyaluronan and that are secreted by some metastatic cells [137–139]. However, these vitamin C analogs themselves degraded hyaluronan, but saccharic acid, another vitamin C analog, inhibited testicular hyaluronidase, but it did not affect physicochemical properties and stability of hyaluronan. Therefore, hyaluronan-related anticancer effects of vitamin C are unlikely to be induced by the natural compound present in the diet.

Triple-negative breast cancer (TNBC), negative for the estrogen and progesterone receptors as well as tyrosine-protein kinase erbB-2 (HER2) receptor, does not respond to hormonal or HER2-targeted therapy and has a high metastatic potential and narrow therapeutic window, so treatment innovations for TNBC are needed [140]. Mustafi et al. showed that vitamin C synergistically improved the efficacy of bromodomain and extraterminal inhibitors (BETi) *in vitro* and *in vivo* [141]. Vitamin C upregulated histone deacetylase 1 (HDAC1) resulting in the suppression of acetylation of H3 and H4 histones. Oral supplementation with vitamin C inhibited metastasis of the human TNBC xenograft in mice.

Anaplastic thyroid carcinoma (ATC) is another type of cancer difficult to cure that accounts for the majority of deaths from thyroid carcinoma [142]. Vitamin C in combination with juglone (5-hydroxy-1,4-naphthalenedione) was shown to inhibit migration, invasion, and angiogenesis in a cell line derived from the ATC ARO cell line, thereby disturbing epithelial-mesenchymal transition suggesting antimetastatic properties of that combination in ATC. It was observed that vitamin C and juglone destabilized the redox balance by affecting catalase, glutathione reductase, and superoxide dismutase indicating prooxidant activity of vitamin C, and its plasma concentration (1 mM) excluded a dietary application to achieve these effects.

## 9. Conclusions and Perspectives

Human epidemiological studies and clinical trials do not determine the role of vitamin C supplementation in cancer prevention and therapy. Many molecular studies suggest that vitamin C can act at least in two opposite ways: anti- and prooxidant. Therefore, the dose-effect relationship for this vitamin can be a continuous function, e.g., a U- or J-shaped association between vitamin C intake and cancer risk. However, information on doses of vitamin C in cohort studies is provided by an ever/never-type questionnaire.

Anticancer therapy can be applied with many compounds, and it is not surprising that the interaction of vitamin C with some of them results in a negative therapeutic outcome. Zou et al. showed that vitamin C inactivated PS-431 (bortezomib) that had been approved for the treatment of multiple myeloma [143]. This inactivation resulted from a direct interaction between vitamin C and PS-431, resulting in the abrogation of the PS-341-induced G2/M arrest, apoptosis, and inhibition of the proteasome. Therefore, it is important to check the possibility of vitamin C to interact with other chemicals used in cancer therapy, especially when multiple chemotherapeutic agents are administered, as in hematopoietic cancers.

In their breakthrough study, Yan et al. confirmed that vitamin C rapidly oxidized in culture media. Similar effect must have occurred in transgenic mice these authors used. However, the question where and how circulating vitamin C was oxidized *in vivo* was not addressed in that study [144]. The ability to control oxidation of vitamin C to DHA is essential for its therapeutic use and requires further work.

Anticancer therapy with high doses of vitamin C requires patients to visit the hospital frequently for several weeks, but in fact, little is known about actual concentration of this vitamin after injection. Nielsen et al. showed that the peak concentration of vitamin C in plasma of metastatic prostate cancer patients was 20.3 mM after intravenous infusion of 60 g of the vitamin with the elimination time less than 2 h [145]. These authors concluded that it was not possible to maintain the concentration of vitamin C in the potential cytotoxic range after termination of infusion, and they proposed a regimen with bolus loading. Welsh et al. observed an almost 100% increase (83 vs. 44 *μ*M) in the plasma ascorbate concentration in nine subjects with pancreatic cancer receiving twice-weekly intravenous 15-125 g ascorbate concurrently with gemcitabine [146]. Similar results were obtained by Monti et al. [147]. Hoffer et al. reported plasma concentration of ascorbate up to 14 mM in 14 patients with various cancers infused with 0.6 g/kg vitamin [148]. Wang et al. observed plasma ascorbate concentration up to 3 mM in thirty-six gastrointestinal patients receiving once daily 0.2-1.5 g/kg, 3 h infusion with ascorbate up to 3 days [149].

These problems belong to fundamental aspects of anticancer therapy with high doses of vitamin C that were recently addressed in an excellent review by Carr and Cook [6]. These authors concluded that cancer patients had lower concentration of vitamin C than healthy control, intravenous infusion is the optimal route of administration of high doses of this vitamin, and this is a patient-safe procedure. However, several questions including optimal doses and the dosing regime as well as interference with other chemotherapeutic agents still wait for the answers.

Many aspects of the role of vitamin C in cancer have not been addressed in this review. They are, among others, microorganism-related cancers—individuals with potentially carcinogenic *H. pylori* infection have lower levels of vitamin C in both gastric acid and serum, so the vitamin may play a role in *H. pylori* prevention and eradication (reviewed in [150]). Vitamin C could enhance anticancer action of several chemotherapeutics, but the vitamin or its analogs may abrogate drug resistance as it was shown for DHA at 1 and 2 mM in lung cancer cells resistant to EGFR- (epidermal growth factor receptor-) targeted therapy [151]. Vitamin C may also play a role in supportive care in cancer patients (reviewed in [152]).

In summary, some hospital-based case-control studies, research on an animal model of vitamin C deficiency, and experimental works on molecular levels suggest that pharmacological doses of vitamin C may exert anticancer effects underlined by several different mechanisms (Table 1). Dietary intervention with vitamin C seems to help in fighting cancer mainly in the cases of its pronounced deficiency in cancer tissue.

Anticancer effects of dietary vitamin C are largely nonspecific as it acts as other low molecular weight antioxidants. Many anticancer mechanisms observed *in vitro* and in animal models should be validated in well-designed clinical trials for different cancers.

## Figures and Tables

**Table 1:** Anticancer effects induced by dietary (D) or pharmacological (P) concentrations of vitamin C or its derivatives. Abbreviations are explained in the main text. Question marks indicate values that are interpolated from experimental data.

Cancer	Effect	Mechanism	Concentration	Reference
Many human cancer cell lines	Cell death	Cu(I)- or Fe(II)-dependent H_2_O_2_ formation, oxidative stress	P	6-10

CRC with KRAS or BRAF mutations, human cell lines, and mouse xenografts	Cell death	Inactivation of GAPDH, depletion of glutathione, ROS increase, decreased ATP, energetic crisis	P	75

AML human cell line	Apoptosis, inhibition of proliferation	ERK phosphorylation, Raf1/MAPK inhibition	P/D(?)	57

Breast or colon human cell line	Cell death	Changes in metabolomic profile, NAD deficiency	P	79

Gastric cancer patients, cell lines	Inhibition of proliferation	Selective upregulation of TMEFE2	D	81

Many cancer cell lines	Killing cancer cells by synergistic or additive action with anticancer drugs, including bleomycin, sorafenib, and auranofin	DNA double-strand break induction; modification of redox balance	P/D	85-87

All cancers	Killing cancer cells by stimulation of the immune system	Fas-induced apoptosis, reduction of the activity of caspase-3, caspase-8, and caspase-10, reduced ROS levels, and increased permeability of the mitochondrial membrane as well as HIF-1/2 activation in monocytes; NK stimulation by IFN-*γ* activation	P/D	3, 93-96

Colorectal cancer	Killing cancer cells by targeting their epigenetic profile	Synergistic or additive effects with DNA-demethylating drugs, TET2 activation	P/D	103

All cancers	Tumor inhibition by eradication of cancer stem-like cells	Synthetic lethality with targeting glycolysis; TET2 activation; TET1 inhibition; oxidative DNA damage; activation of the MLH1/c-Abl/p73 signaling; inhibition of pluripotency factors, incl. OCT4A, SOX2, and NANOG	P/D(?)	114-121

All cancers	Metastasis inhibition	Inhibition of MMP secretion; inhibition of hyaluronidase and hyaluronan lyase; increasing efficacy of BET1; upregulation of HDAC1; inhibition epithelial-mesenchymal transition	P/D	127-137
